# Exercise, vagal regulation, and inflammatory symptom clusters in breast cancer survivors: a structured mini-review and hypothesis-generating framework

**DOI:** 10.3389/fpsyg.2026.1872288

**Published:** 2026-06-10

**Authors:** Yulong Feng, Jinhu Wei

**Affiliations:** Guangxi Science and Technology Normal University, Laibin, China

**Keywords:** breast cancer survivorship, cancer-related fatigue, cholinergic anti-inflammatory pathway, exercise, heart rate variability, inflammation, psycho-oncology rehabilitation, symptom clusters

## Abstract

Breast cancer survivors frequently experience co-occurring fatigue, sleep disturbance, anxiety, depressive symptoms, and pain. These symptoms may partly reflect a shared psychophysiological state involving autonomic dysregulation, impaired stress recovery, and low-grade inflammatory activity. Exercise is increasingly recommended as a supportive care strategy for cancer-related fatigue and functional recovery, but the psychophysiological mechanisms linking exercise to symptom improvement remain incompletely understood. This structured mini-review proposes a hypothesis-generating framework in which heart rate variability (HRV), particularly vagally mediated indices such as the root mean square of successive differences (RMSSD) and high-frequency HRV (HF-HRV), may represent a candidate indicator of autonomic flexibility linking exercise, inflammatory activity, and symptom clustering in breast cancer survivors. The cholinergic anti-inflammatory pathway and α7 nicotinic acetylcholine receptor (α7nAChR) signaling are discussed as biologically plausible neuroimmune mechanisms, but direct evidence for this pathway in breast cancer survivorship remains limited. Exercise-related changes in HRV may be consistent with improved vagal regulation and inflammatory moderation; however, HRV is influenced by multiple clinical and behavioral confounders, including cancer treatment exposure, medication use, sleep, comorbidities, and baseline fitness. Therefore, HRV should be interpreted as an adjunctive psychophysiological marker rather than as direct evidence of a causal mechanism. Future trials should combine standardized HRV monitoring, inflammatory biomarkers, and validated symptom-cluster outcomes to test whether changes in autonomic regulation mediate the effects of exercise on inflammation and symptom burden in breast cancer survivors.

## 1 Introduction

The long-term psychophysiological condition of breast cancer survivorship is increasingly recognized as being shaped by treatment exposure, endocrine disruption, stress adaptation, and persistent symptom burden. Cancer-related fatigue, sleep disturbances, symptoms of depression and anxiety, pain, and cognitive problems are among the most debilitating side effects, often co-occurring rather than appearing in isolation (Bower, 2008; Bower, 2014; Bower et al., 2011). This clustering is clinically significant because the symptoms exacerbate each other: Poor sleep may heighten fatigue and sensitivity to pain, while fatigue decreases physical activity and social interactions, and emotional distress may further hinder recovery. Hence, survivorship challenges are not just about tumor status or finishing treatment; they also involve the survivor’s capacity to restore neuroimmune, autonomic, and behavioral balance.

A growing body of research suggests that these symptom clusters may be partly linked to inflammatory biology. Pro-inflammatory signals, including interleukin-6, tumor necrosis factor-*α*, interleukin-1β, and C-reactive protein, have been associated with fatigue, depression, sleep disturbances, and pain in cancer patients (Bower, 2014; Bower et al., 2011; Doong et al., 2015; Myers, 2008). In breast cancer, post-treatment behavioral symptoms have been associated with inflammatory activity, whereas cytokine-related genetic variations have been associated with the coexistence of pain, fatigue, sleep disturbances, and depression prior to surgery (Bower et al., 2011; Doong et al., 2015). These findings do not indicate that inflammation is the sole cause of survivorship symptoms. They argue for a more nuanced viewpoint: low-grade inflammation may act as a biological amplifier, connecting peripheral immune activation to central motivational, affective, and sleep-regulatory systems.

This inflammatory viewpoint is particularly relevant to psychophysiology, as immune activity is not governed in isolation. The autonomic nervous system acts as a bidirectional interface between psychological stress, cardiovascular regulation, and immune function. Heart rate variability (HRV), specifically indices of parasympathetic modulation such as RMSSD and high-frequency HRV (HF-HRV), is a non-invasive indicator of this interaction. Reduced HRV has been linked to cancer-related fatigue in breast cancer survivors, and meta-analyses of human studies show an inverse relationship between HRV and inflammatory markers (Crosswell et al., 2014; Williams et al., 2019). HRV should not be interpreted as a direct or exclusive measure of vagal tone. Rather, vagally mediated indices such as RMSSD and HF-HRV provide indirect information about parasympathetic cardiac modulation under standardized measurement conditions. In breast cancer survivorship, HRV may be useful as a candidate indicator of autonomic flexibility, but its interpretation requires attention to treatment exposure, medication use, cardiovascular status, sleep, respiration, and baseline fitness.

The vagus nerve is central to this framework. In addition to its role in cardiac regulation, vagal signaling participates in the inflammatory reflex through the cholinergic anti-inflammatory pathway. Acetylcholine-induced activation of α7 nicotinic acetylcholine receptors on immune cells inhibits pro-inflammatory cytokine production, linking neural activity to immune suppression (Pavlov and Tracey, 2005; Báez-Pagán et al., 2015). This mechanism provides a biologically plausible link between psychological distress, autonomic dysregulation, and inflammatory symptoms in breast cancer survivors. Vagal withdrawal may be associated with reduced anti-inflammatory regulation, whereas improved vagal regulation may accompany physiological recovery.

Exercise is well positioned to target this dimension. Current clinical guidelines recommend exercise as a primary non-pharmacological approach to cancer-related fatigue, and systematic evidence suggests that exercise may improve autonomic modulation in cancer patients and survivors (Bower et al., 2024; Lavín-Pérez et al., 2021). However, the mechanistic interpretation of exercise in survivorship care is frequently broad, defined in terms of improved fitness, mood, or overall inflammation. This mini-review takes a more focused psychophysiological approach. We propose that exercise may be associated with symptom improvement partly through changes in autonomic regulation and inflammatory activity. In this framework, HRV is considered not only as a physiological outcome but also as a candidate psychophysiological indicator that may help characterize relationships among exercise exposure, autonomic regulation, inflammatory activity, and symptom outcomes, whereas vagus-mediated anti-inflammatory signaling is discussed as a plausible but insufficiently validated pathway requiring direct testing in breast cancer survivor cohorts.

**Figure 1:**
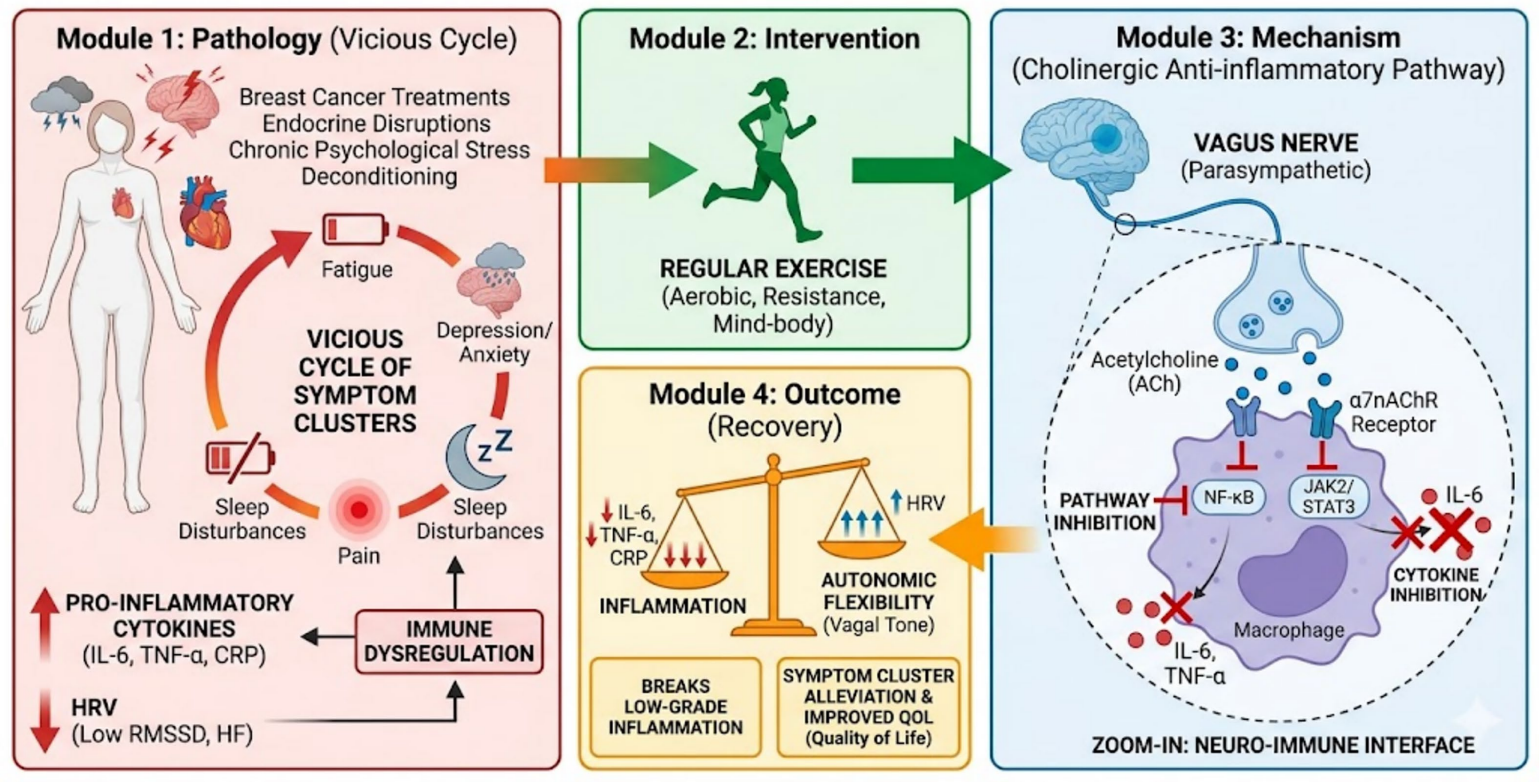
Proposed psychophysiological framework linking exercise, HRV, vagal regulation, inflammatory activity, and symptom clusters in breast cancer survivors.

This mini-review proposes a hypothesis-generating framework focused on the exercise-HRV/vagal tone-inflammation-symptom cluster pathway. We first discuss the significance of inflammatory symptom clusters in breast cancer survivorship, then examine HRV and vagal regulation as psychophysiological indicators, and finally outline how exercise-related autonomic changes may be linked to inflammatory activity and symptom outcomes. The novelty of this review lies in integrating three areas that are often discussed separately: autonomic dysfunction in breast cancer survivorship, inflammatory symptom clustering, and exercise-based rehabilitation. Previous reviews have examined HRV abnormalities in breast cancer patients and survivors, and other work has summarized exercise effects on fatigue, inflammation, or physical function. In contrast, this mini-review conceptualizes HRV as a candidate regulatory node within a broader exercise-autonomic-inflammatory-symptom framework. The α7nAChR-related cholinergic anti-inflammatory pathway is not presented as an established mechanism in breast cancer survivors, but as a biologically plausible neuroimmune interface that may help generate testable hypotheses for future exercise-oncology trials. Figure 1 summarizes the proposed psychophysiological framework. Breast cancer treatment exposure, psychological stress, endocrine disruption, sleep disturbance, and physical deconditioning may contribute to autonomic imbalance and low-grade inflammatory activity. These processes may reinforce fatigue, mood symptoms, sleep problems, and pain. Exercise is proposed as a behavioral intervention that may improve autonomic flexibility and potentially reduce inflammatory symptom burden. Dashed arrows indicate hypothesized links that require direct validation in breast cancer survivor cohorts.

Breast cancer treatment exposure, psychological stress, endocrine disruption, sleep disturbance, and physical deconditioning may contribute to reduced HRV, autonomic imbalance, and low-grade inflammatory activity. These processes may reinforce co-occurring fatigue, depressive and anxiety symptoms, sleep disturbance, and pain. Regular exercise, including aerobic, resistance, and mind–body exercise, is proposed to improve autonomic flexibility and may contribute to inflammatory moderation. The enlarged neuroimmune interface illustrates the hypothesized cholinergic anti-inflammatory pathway, in which acetylcholine signaling through α7nAChR may inhibit inflammatory signaling pathways such as NF-κB and JAK2/STAT3 and reduce pro-inflammatory cytokine production. Dashed arrows indicate hypothetical relationships requiring direct validation in breast cancer survivors.

## 2 Review methodology

This mini-review used a structured narrative search strategy to identify literature relevant to exercise, autonomic regulation, inflammation, and symptom clusters in breast cancer survivorship. Searches were conducted in PubMed/MEDLINE, Web of Science, Scopus, and PsycINFO, with Google Scholar used for supplementary citation tracking. The search covered publications from January 2000 to March 2026. Search terms included combinations of “breast cancer survivor,” “breast cancer survivorship,” “exercise,” “physical activity,” “heart rate variability,” “HRV,” “vagal tone,” “vagal regulation,” “autonomic regulation,” “inflammation,” “cytokines,” “IL-6,” “TNF-*α*,” “CRP,” “symptom cluster,” “fatigue,” “sleep disturbance,” “depression,” “anxiety,” “pain,” “cholinergic anti-inflammatory pathway,” and “α7 nicotinic acetylcholine receptor.”

Eligible literature included original human studies, clinical trials, observational studies, systematic reviews, meta-analyses, and mechanistic papers relevant to the proposed exercise-HRV-inflammation-symptom framework. Studies were prioritized if they involved breast cancer patients or survivors; cancer survivor studies and non-oncology exercise-HRV studies were included when breast cancer-specific evidence was limited and when they provided relevant mechanistic or methodological context. Studies were excluded if they were unrelated to cancer survivorship, did not address exercise, autonomic regulation, inflammation, or symptom outcomes, or were conference abstracts without full-text data.

Titles and abstracts were screened for relevance, followed by full-text review of studies directly related to the proposed framework. Reference lists of key papers were also examined to identify additional relevant sources. Because the purpose of this article was to develop a hypothesis-generating psychophysiological framework rather than to conduct a systematic review, formal risk-of-bias assessment and quantitative synthesis were not performed. The last search was conducted on March 30, 2026. After screening titles and abstracts, studies were selected based on relevance to at least one component of the proposed framework: breast cancer survivorship, exercise, HRV or autonomic regulation, inflammation, symptom clusters, or cholinergic anti-inflammatory signaling.

## 3 Findings

### 3.1 Heart rate variability and vagal tone as psychophysiological indicators

#### 3.1.1 HRV as an indicator of autonomic flexibility

HRV refers to the oscillation in cardiac interbeat intervals caused by sympathetic and parasympathetic influences on the sinoatrial node (Task force of the European Society of Cardiology and the North American Society of Pacing and Electrophysiology, 1996; Shaffer and Ginsberg, 2017). While HRV is often used as a cardiovascular metric, its conceptual significance in cancer survivorship is much broader. The heart is constantly influenced by neural systems that coordinate physiological arousal, emotional significance, cognitive regulation, and inflammatory responses. HRV provides non-invasive information about the organism’s ability to adapt to internal and external demands.

Among HRV indices, RMSSD and HF-HRV are commonly regarded as indices of short-term parasympathetic, or vagally mediated cardiac modulation, whereas SDNN reflects broader variability over the recording duration (Task force of the European Society of Cardiology and the North American Society of Pacing and Electrophysiology, 1996; Shaffer and Ginsberg, 2017). This distinction is critical for psychophysiological research because vagal influences can quickly alter cardiac output in response to stress, recovery, respiration, and emotional regulation. Elevated resting vagally mediated HRV in this context should not be interpreted simply as a “relaxed” state; it more accurately represents autonomic flexibility—the ability to mobilize physiological resources as needed and efficiently return to a state of recovery following a challenge.

The neurovisceral integration model provides an important theoretical foundation for this interpretation. According to this framework, HRV reflects the functional connectivity of the prefrontal, limbic, and brainstem circuits that govern cognitive control, emotional regulation, and autonomic responses (Thayer and Lane, 2000; Thayer et al., 2009). Lower HRV may thus suggest a less adaptable regulatory system in which emotional distress, impaired stress recovery, and physiological dysregulation all reinforce one another. This perspective is especially important for breast cancer survivors, as treatment-related stressors, endocrine disruption, deconditioning, sleep disturbances, and ongoing symptom burden may all have an impact on autonomic regulation.

HRV has been studied in breast cancer cohorts as a measure of cardiac autonomic regulation and susceptibility to survivorship. A systematic review of breast cancer patients and survivors found that HRV analysis could provide clinically relevant insights into autonomic dysfunction in this population (Arab et al., 2016). Lower HRV has been linked to cancer-related fatigue in young breast cancer survivors, suggesting that autonomic dysregulation may contribute to the persistence of symptoms after primary treatment (Crosswell et al., 2014). This mini-review considers HRV not only as a physiological outcome, but also as a candidate psychophysiological indicator that may help characterize the relationship among exercise exposure, autonomic regulation, inflammatory activity, and symptom outcomes.

#### 3.1.2 Vagal withdrawal, psychological distress, and inflammation: associations rather than established causality

Vagal withdrawal is a plausible psychophysiological mechanism through which psychological distress and inflammatory activity may be linked in breast cancer survivorship. The vagus nerve is involved in both cardiac regulation and immune modulation. Reduced vagal tone may result in decreased parasympathetic recovery, increased sympathetic dominance, and impaired regulation of inflammatory responses. This regulatory profile may increase susceptibility to fatigue, anxiety, depressive symptoms, sleep disturbances, and pain.

Psychophysiological evidence supports this interpretation. Meta-analytic evidence suggests that depression and anxiety disorders are associated with decreased heart rate variability, implying that affective symptoms frequently coexist with reduced autonomic flexibility (Kemp et al., 2010; Chalmers et al., 2014; Wang et al., 2023). In oncology, preoperative anxiety and depression have been associated with altered heart rate variability in breast cancer patients, implying that emotional distress may correlate with quantifiable autonomic dysfunction (Farbood et al., 2020). A recent cross-sectional study of breast cancer patients found that decreased HRV was associated with increased inflammatory activity and anxiety, supporting the possibility that HRV may reflect a comprehensive autonomic-immune-affective phenotype rather than just a cardiac signal (Zhang et al., 2025).

The inflammatory aspect of HRV is supported by extensive human evidence. A meta-analysis found a negative correlation between HRV and inflammatory markers, with SDNN and HF-HRV having particularly strong associations with inflammatory activity (Williams et al., 2019). This relationship is biologically plausible because the autonomic nervous system participates in the inflammatory reflex, a neural circuit in which vagal signaling influences cytokine production (Thayer and Sternberg, 2009). According to this viewpoint, decreased HRV may be consistent with impaired vagal anti-inflammatory regulation, in which psychological distress and inflammatory symptoms become increasingly difficult to distinguish.

This is significant in the context of breast cancer survivorship because fatigue, depressive mood, anxiety, sleep disturbances, and pain are rarely experienced alone. They frequently form patterns similar to sickness behavior biology, in which inflammatory signaling influences motivation, energy availability, sleep architecture, and emotional tone. Vagal withdrawal may act as a regulatory constraint: while it may not directly cause every symptom, it can impair the system’s ability to deal with inflammatory and psychological disturbances. HRV is particularly appealing for exercise-based supportive care because it is measurable, modifiable, and serves as a candidate regulatory marker between behavioral intervention and neuroimmune regulation.

Nonetheless, caution is essential. HRV is more than just a measure of vagal tone; it can also be influenced by respiration, posture, circadian rhythms, medication, age, cardiovascular comorbidities, physical fitness, and cancer treatments (Task force of the European Society of Cardiology and the North American Society of Pacing and Electrophysiology, 1996; Shaffer and Ginsberg, 2017; Arab et al., 2016). Furthermore, current breast cancer research has yet to identify a comprehensive causal pathway that links vagal withdrawal to inflammation and symptom aggregation. The most convincing interpretation is that HRV does not, by itself, explain cancer-related symptoms, but rather acts as an indicator of a psychophysiological regulatory state that connects exercise, stress recovery, and inflammatory symptom burden.

The available evidence supports an association among lower HRV, psychological distress, and inflammatory activity, but it does not establish a unidirectional causal pathway in breast cancer survivors. Psychological symptoms, inflammatory activation, cancer treatment exposure, and physical deconditioning may interact bidirectionally. Therefore, HRV is best understood as a candidate marker of regulatory state rather than a definitive causal mediator of symptom burden.

### 3.2 Exercise and the vagal-inflammatory axis: proposed associations and mechanistic hypotheses

#### 3.2.1 Exercise-related changes in vagal regulation

For breast cancer survivors, exercise may be beneficial in managing inflammatory symptom clusters by improving their fitness levels and partly supporting autonomic flexibility. In this context, vagal regulation is a relevant psychophysiological target due to its association with cardiovascular adaptation, stress recovery, and immune regulation. Autonomic balance can be disrupted by cancer treatment, physical deconditioning, sleep disturbances, and ongoing psychological stress, leading to increased sympathetic activity and reduced vagal tone. Consistent exercise may mitigate this trend in some survivors by improving cardiorespiratory fitness, supporting parasympathetic reactivation, and potentially increasing vagally mediated HRV indices such as RMSSD and HF-HRV. However, exercise-induced autonomic changes are not uniform. Training intensity, recovery status, treatment-related cardiotoxicity, medication use, sleep quality, and baseline fitness may influence whether HRV improves, remains unchanged, or temporarily decreases during an intervention (Lavín-Pérez et al., 2021; Toohey et al., 2020; Amekran and El Hangouche, 2024).

Although the evidence in cancer populations varies, it supports the biological plausibility of this pathway. A systematic review and meta-analysis found that in cancer patients and survivors, exercise interventions may improve autonomic regulation related to HRV (Lavín-Pérez et al., 2021). A pilot study of breast cancer survivors found that the improvement in cardiovascular fitness and cardiac regulation markers after high-intensity interval training suggest that structured exercise can impact autonomic adaptation post-cancer treatment (Toohey et al., 2020). Randomized trial evidence in adults shows that exercise training can enhance heart rate variability metrics linked to vagal activity, suggesting that physical activity can modify autonomic regulation beyond just boosting subjective well-being.

For breast cancer survivors, this autonomic effect could be clinically relevant because a decrease in vagal tone can heighten the physiological stress load. A reduction in HRV might suggest difficulties in recovering from emotional or physical stress, potentially causing extended fatigue, sleep disturbances, and mood-related symptoms. By enhancing the organism’s ability to alternate between activation and recovery, exercise may help disrupt this cycle. Cardiorespiratory fitness and parasympathetic reactivation are particularly influenced by aerobic exercise, while resistance training might support autonomic recovery by enhancing muscle function, metabolic health, and reducing fatigue resistance. Mind–body exercises like yoga, Tai Chi, and breathing-based movements could be especially useful for modulating vagal regulation, as they merge moderate physical effort with respiratory control, focus regulation, and stress relief.

Exercise should not be viewed as a uniform autonomic stimulus. High intensity, insufficient recovery, or treatment-related vulnerability may temporarily lower HRV, while well-adjusted training can enhance autonomic resilience over time. This distinction is critical to a psychophysiological framework for exercise prescription. The therapeutic goal is not maximal exertion, but HRV may indicate changes in autonomic recovery and may provide useful feedback for adjusting exercise dose in breast cancer survivorship.

#### 3.2.2 Cholinergic anti-inflammatory signaling and α7nAChR: a plausible but insufficiently validated pathway

The anti-inflammatory relevance of vagal regulation is often discussed in relation to the inflammatory reflex and the cholinergic anti-inflammatory pathway. In this pathway, acetylcholine signaling can suppress pro-inflammatory cytokine production by immune cells, partly through α7nAChR-related mechanisms (Pavlov and Tracey, 2005; Thayer and Sternberg, 2009; Gallowitsch-Puerta and Pavlov, 2005). However, direct evidence that exercise modulates α7nAChR signaling in breast cancer survivors is currently scarce. Therefore, this pathway should be interpreted as a biologically plausible hypothesis rather than an established mechanism of exercise-induced symptom improvement in oncology populations (Wang et al., 2003).

Activation of α7nAChR may reduce inflammatory signaling through inhibition of the NF-κB and JAK2-STAT3 pathways, which limits the synthesis of cytokines such as TNF-*α*, IL-1β, and IL-6 (Báez-Pagán et al., 2015; Wang et al., 2003). These cytokines are connected to fatigue, depressive symptoms, sleep problems, and pain sensitivity, making this mechanism relevant to the biology of cancer-related symptoms. Within the proposed model, exercise-related changes in vagal regulation may be consistent with improved inflammatory regulation, but whether this involves α7nAChR-related signaling in breast cancer survivors remains unproven.

Direct evidence linking exercise to α7nAChR signaling is limited in breast cancer survivors. Nonetheless, several lines of indirect evidence support this hypothesis. First, exercise has been shown to improve autonomic regulation and heart rate variability mediated by the vagus nerve in cancer patients (Lavín-Pérez et al., 2021; Toohey et al., 2020). Evidence suggests that exercise training may decrease inflammatory biomarkers like CRP, IL-6, and TNF-*α* in those with breast cancer and survivors (Abbasi et al., 2022; Bettariga et al., 2025). Research on human exercise outside of oncology suggests that different methods might impact the expression of CHRNA7 and CHRFAM7A in leukocytes, suggesting that physical activity can alter molecular components linked to α7nAChR-mediated immune control (Mateus et al., 2023). These findings support the hypothesis that exercise-related changes in symptoms and inflammation may be partly linked to vagal regulation and α7nAChR-associated anti-inflammatory pathways, although direct evidence in breast cancer survivors remains limited.

This hypothesis should be interpreted with caution. While exercise may be associated with changes in HRV, cytokine activity, and symptom outcomes, direct evidence that these effects are mediated through α7nAChR-related signaling in breast cancer survivors is lacking. A study combining exercise intervention, HRV monitoring, along with inflammatory biomarkers and symptom clusters, might determine if alterations in vagally mediated HRV statistically mediate exercise’s effects on inflammation, fatigue, mood, or sleep. This framework may help shift exercise oncology from general statements about ‘reducing inflammation’ to a more detailed psychophysiological model of supportive care.

### 3.3 Proposed framework and prospective pathways

#### 3.3.1 A cluster framework linking exercise, HRV, inflammation, and symptoms

We propose a model that links exercise, heart rate variability, inflammation, and symptom clusters to conceptualize how physical activity may influence long-term symptom burden in breast cancer survivors. This framework depicts the impact of breast cancer treatment, chronic psychological stress, sleep disruption, endocrine therapy, and physical deconditioning on autonomic control. The resulting state may be characterized by decreased vagally mediated heart rate variability, impaired physiological recovery, and a shift to low-grade inflammatory activity. This autonomic-inflammatory condition may amplify fatigue, anxiety, depressive symptoms, sleep disturbances, and pain, resulting in a self-perpetuating cluster of symptoms rather than a collection of separate issues.

Exercise may be relevant to this framework at multiple levels. First, consistent exercise can improve cardiorespiratory fitness and reduce physiological stress caused by daily activities. Second, exercise may improve autonomic flexibility, as evidenced by increased heart rate variability and better parasympathetic reactivation after stress. Third, exercise-related improvements in vagal regulation may be hypothesized to influence cholinergic anti-inflammatory signaling and inflammatory tone associated with cancer-related symptoms, although this pathway remains insufficiently validated. This model aligns with exercise oncology guidelines, which recommend exercise as a safe and effective intervention for cancer-related fatigue and physical functioning. It also supports evidence that exercise can influence both heart rate variability and inflammatory biomarkers in cancer populations (Bower et al., 2024; Lavín-Pérez et al., 2021; Abbasi et al., 2022; Bettariga et al., 2025; Campbell et al., 2019).

The model is deliberately integrative rather than deterministic. HRV is not identified as the sole cause of inflammatory symptoms, nor is inflammation thought to be the sole biological basis of fatigue or emotional distress. HRV is conceptualized as a candidate regulatory marker that may help integrate behavioral, autonomic, and immune processes. In this context, decreased HRV may indicate a reduction in adaptive capacity, whereas an increase in HRV following exercise may indicate a partial restoration of psychophysiological resilience.

One important implication is that symptom relief after exercise should not be viewed solely as a result of improved fitness or mood. This may signal a shift in the regulatory state, from sympathetic dominance, vagal withdrawal, and inflammatory enhancement to increased autonomic flexibility and immune moderation. This interpretation is especially useful for breast cancer survivorship, where symptoms often persist after primary treatment and recovery mechanisms are not solely explained by tumor-related factors.

#### 3.3.2 Enhancing HRV-informed exercise prescription for breast cancer survivors

Furthermore, the proposed model supports further exploration of HRV-guided exercise prescription. Standard oncology exercise prescriptions typically take into account frequency, intensity, duration, and type, as well as adjustments for treatment status, comorbidities, and functional capacity (Campbell et al., 2019). This approach is clinically useful, but it may not fully capture daily fluctuations in autonomic recovery, fatigue levels, and physiological preparedness. HRV may help refine exercise prescription by helping clinicians estimate whether a given exercise dosage promotes adaptation or contributes to excessive physiological strain.

In practice, HRV-guided exercise prescription should not replace standard clinical evaluation. Instead, it could complement symptom assessments, performance evaluations, and inflammatory biomarkers. Consistently low RMSSD or low HF-HRV, combined with significant fatigue or insufficient sleep, may suggest the need for lower-intensity aerobic exercise, breath-focused movement, yoga, or Tai Chi. In contrast, stable or improved HRV combined with symptom relief may support a gradual transition to integrated aerobic and resistance training. Data from non-cancer cohorts show that HRV-guided training may improve vagally mediated HRV and reduce adverse responses when compared to fixed training protocols, though this methodology is still in its early stages in oncology (Manresa-Rocamora et al., 2021). Initiatives at the protocol level have begun to investigate HRV-based exercise prescriptions for breast cancer patients undergoing chemotherapy; however, definitive evidence is still lacking (Lavín-Pérez et al., 2023).

Subsequent research should evaluate the proposed pathway using longitudinal and mechanistically informed intervention designs. An ideal trial would combine a structured exercise intervention with ongoing HRV monitoring, inflammatory biomarkers, and validated symptom-cluster outcomes. Mediation analyses could explore how changes in vagally mediated heart rate variability contribute to the link between exercise and lower levels of IL-6, TNF-*α*, CRP, fatigue, sleep disturbances, and affective symptoms. Such research would help determine whether HRV is simply a predictor of recovery or a useful adjunctive marker for tailoring exercise-based supportive care.

Several methodological issues will be important. HRV must be evaluated under standardized conditions, which include standardized posture, time of day, recording duration, respiratory control or documentation, medication status, and treatment phase (Task force of the European Society of Cardiology and the North American Society of Pacing and Electrophysiology, 1996; Shaffer and Ginsberg, 2017).

Symptom outcomes should be assessed as clusters rather than discrete endpoints, as fatigue, sleep disturbances, pain, and mood symptoms frequently interact. Inflammatory biomarkers should be interpreted with caution, preferably using repeated measurements to account for biological variability. Ultimately, future research should avoid overextending tumor-related interpretations. The primary benefit of this framework is that it provides supportive care, which improves symptom management, recovery potential, and quality of life for breast cancer survivors.

### 3.4 Methodological considerations and confounders

Interpretation of HRV and inflammatory biomarkers in breast cancer survivors requires careful attention to confounding factors. Chemotherapy-induced cardiotoxicity, particularly after anthracycline or trastuzumab exposure, may affect cardiac autonomic regulation independently of exercise. Radiotherapy involving the left chest wall, endocrine therapy, menopausal status, obesity, metabolic dysfunction, cardiovascular comorbidities, and baseline physical activity may also influence HRV and inflammatory markers. Medication use is another major concern. Sleep medications, antidepressants, beta-blockers, analgesics, anti-inflammatory drugs, and endocrine agents may alter autonomic or inflammatory profiles.

Short-term HRV measurements are additionally sensitive to posture, respiration, circadian timing, caffeine intake, recent physical exertion, sleep quality, pain flares, infection, and acute psychological stress. These factors may obscure whether observed HRV changes reflect exercise adaptation, treatment-related autonomic injury, medication effects, or broader health status. Future intervention studies should therefore standardize HRV acquisition protocols, record cancer treatment history and medication use, stratify or adjust for menopausal and cardiometabolic status, and interpret HRV alongside symptom ratings, physical function, and inflammatory biomarkers.

### 3.5 Clinical implications for HRV-informed exercise monitoring

HRV-informed exercise monitoring should not replace standard clinical assessment or established exercise oncology guidelines. Its potential value lies in complementing symptom ratings, perceived exertion, sleep quality, treatment status, and functional capacity. For example, persistently reduced RMSSD or HF-HRV together with severe fatigue, poor sleep, or elevated perceived exertion may suggest the need for lower-intensity aerobic activity, breathing-based exercise, yoga, Tai Chi, or additional recovery. In contrast, stable or improving HRV together with symptom relief and good exercise tolerance may support gradual progression toward combined aerobic and resistance training.

In psycho-oncology rehabilitation programs, HRV could be used as a longitudinal monitoring tool rather than a one-time diagnostic measure. Repeated measurements under standardized conditions may help clinicians identify periods of inadequate recovery, excessive training load, or vulnerability to symptom exacerbation. However, HRV-guided prescription in breast cancer survivorship remains experimental. Clinical implementation should therefore be cautious, individualized, and integrated with patient-reported outcomes and medical evaluation.

## 4 Conclusion

Breast cancer survivors frequently report co-occurring fatigue, sleep disturbance, anxiety, depressive symptoms, and pain. These symptoms should not be dismissed as purely psychological complaints or nonspecific treatment sequelae. They may partly reflect a psychophysiological state in which autonomic dysregulation, impaired stress recovery, and low-grade inflammatory activity interact to maintain symptom clustering. HRV, particularly vagally mediated indices such as RMSSD and HF-HRV, may provide a useful candidate indicator of autonomic flexibility, but it should not be interpreted as a direct or isolated measure of vagal tone. This mini-review proposes a hypothesis-generating framework in which exercise may influence symptom burden partly through changes in autonomic regulation and inflammatory activity. The α7nAChR-related cholinergic anti-inflammatory pathway provides a biologically plausible neuroimmune interface, but direct evidence for the complete exercise-HRV-α7nAChR-inflammation-symptom pathway in breast cancer survivors remains limited. For psycho-oncology rehabilitation, this framework suggests that exercise prescription may benefit from integrating physiological recovery markers with conventional symptom and functional assessments. HRV-informed monitoring may help identify periods of inadequate recovery, support individualized adjustment of exercise intensity, and improve tolerability in survivors with persistent fatigue, sleep disturbance, or affective symptoms. Future intervention trials should combine standardized HRV monitoring, inflammatory biomarkers, medication and treatment-exposure data, and validated symptom-cluster outcomes to determine whether changes in autonomic regulation mediate the effects of exercise on inflammation and symptom recovery.
